# Influence of Maternal Immune Activation and Stressors on the Hippocampal Metabolome

**DOI:** 10.3390/metabo13080881

**Published:** 2023-07-25

**Authors:** Bruce R. Southey, Rodney W. Johnson, Sandra L. Rodriguez-Zas

**Affiliations:** 1 Department of Animal Sciences, University of Illinois at Urbana-Champaign, Urbana, IL 61801, USA; rwjohn@illinois.edu (R.W.J.); rodrgzzs@illinois.edu (S.L.R.-Z.); 2 Department of Statistics, University of Illinois at Urbana-Champaign, Urbana, IL 61801, USA

**Keywords:** metabolite, metabolomics, hippocampus, gas chromatography, mass spectrometry, maternal immune activation, immunometabolism

## Abstract

Prenatal stress often results in maternal immune activation (MIA) that can impact prenatal brain development, molecular processes, and substrates and products of metabolism that participate in physiological processes at later stages of life. Postnatal metabolic and immunological stressors can affect brain metabolites later in life, independently or in combination with prenatal stressors. The effects of prenatal and postnatal stressors on hippocampal metabolites were studied using a pig model of viral MIA exposed to immunological and metabolic stressors at 60 days of age using gas chromatography mass spectrometry. Postnatal stress and MIA elicited effects (FDR-adjusted *p*-value < 0.1) on fifty-nine metabolites, while eight metabolites exhibited an interaction effect. The hippocampal metabolites impacted by MIA or postnatal stress include 4-aminobutanoate (GABA), adenine, fumarate, glutamate, guanine, inosine, ornithine, putrescine, pyruvate, and xanthine. Metabolites affected by MIA or postnatal stress encompassed eight significantly (FDR-adjusted *p*-value < 0.1) enriched Kyoto Encyclopedia of Genes and Genomes Database (KEGG) pathways. The enriched arginine biosynthesis and glutathione metabolism pathways included metabolites that are also annotated for the urea cycle and polyamine biosynthesis pathways. Notably, the prenatal and postnatal challenges were associated with disruption of the glutathione metabolism pathway and changes in the levels of glutamic acid, glutamate, and purine nucleotide metabolites that resemble patterns elicited by drugs of abuse and may underlie neuroinflammatory processes. The combination of MIA and postnatal stressors also supported the double-hit hypothesis, where MIA amplifies the impact of stressors later in life, sensitizing the hippocampus of the offspring to future challenges. The metabolites and pathways characterized in this study offer evidence of the role of immunometabolism in understanding the impact of MIA and stressors later in life on memory, spatial navigation, neuropsychiatric disorders, and behavioral disorders influenced by the hippocampus.

## 1. Introduction

Metabolites are integral components of the brain’s immune processes beyond their established bioenergetic or biosynthesis roles. Immune response activation elicits changes in metabolic reprogramming, including shifting energy production pathways and prioritizing other immune-related pathways, including fatty acid synthesis and the production of antibacterial metabolites such as itaconic acid [1,2,3]. Substrates, intermediates, and products in the metabolic pathways, such as adenosine and succinate, can directly modulate the immune response [4,5,6]. Disruption of the amino acid metabolism, particularly phenylalanine in the hippocampus, impairs fear learning and memory [7].

The hippocampus modulates multiple processes, including memory and learning. As part of the limbic system, the hippocampus regulates the function of the hypothalamus and the hypothalamus-pituitary-adrenal (HPA) axis [8]. The hippocampus also acts as a hub where metabolic signals are integrated with cognitive processes [9,10]. Stressors can dysregulate the hippocampus at different stages of development and life [11], and HPA axis dysregulation can result in immune system dysfunction [12]. In response to immune and other challenges, the HPA releases glucocorticoids that provide anti-inflammatory and immunosuppressive actions [13]. Hippocampal neuroinflammation alters glutamatergic neurotransmission, which has been connected with cognitive impairment [14].

Prenatal stressors can elicit maternal immune activation (MIA), and the resulting maternal cytokine and hormonal signals can disrupt the fetal central nervous system [9,15,16,17,18,19]. Among the effects, hippocampal neurogenesis and molecular pathways were particularly sensitive to the maternal signaling milieu [8,11]. Postnatal stressors can disrupt physiological, neural, and molecular processes in the offspring [20]. The joint effects of prenatal and postnatal stressors have been reasoned under the double-hit hypothesis studied in rodent, pig, and primate models [9,21,22,23]. The combination of weaning stress and MIA affects serum indicators [24], metabolomics [25], the amygdala and hippocampus transcriptome [26,27,28,29,30], and the behavior of pigs [31]. MIA and postnatal challenges have [9,21,22,23] been associated with changes in serum indicators and neurodevelopmental disorders, including schizophrenia and autism spectrum disorders in humans [18,19]. 

The direct impact of prenatal and postnatal stressors on the metabolic pathways of brain regions that modulate the HPA axis, memory processes, and feeding and social behaviors is incompletely understood. Previously, there was limited evidence of the double-hit hypothesis in the liver with weaning stress and MIA [25]. The objective of this immunometabolomic study is to advance our understanding of the effects of MIA and stressors on the hippocampal metabolome. Female and male pigs were exposed to MIA elicited by the porcine reproductive and respiratory syndrome virus (PRRSV), a virus from the Nidovirales order that includes coronaviruses. At 60 days of age (after weaning stress but before puberty-related changes) [32], pigs were exposed to the metabolic stress of fasting or an immune challenge elicited by a viral mimetic. Hippocampal metabolic profiles and pathways were examined for the combined impact of MIA and stress at 60 days of age. The analysis of metabolite changes furthers our understanding of the association between the double-hit hypothesis and physiological and behavioral changes. In addition to characterizing immunmetabolism in the hippocampus, this study supports the development of therapeutic strategies to mitigate the effects of MIA and other stressors. 

## 2. Materials and Methods

### 2.1. Animal Experiments

Hippocampi metabolomic profiles were studied in 60-day-old pigs from Camborough gilts (PIC, Hendersonville, TN, USA) that were inseminated with PIC 359 boar semen (PIC, Hendersonville, TN, USA). At gestation day 69, PRRSV-negative gilts were individually housed in identical disease containment chambers maintained at 22 °C with a 12-hour light/dark cycle with lights on at 7:00 a.m. following proving protocols [26,31]. The gilts were randomly assigned into MIA and control groups. At gestation day 76, the MIA group was intranasally inoculated with live PRRSV strain P129-BV (School of Veterinary Medicine at Purdue University, West Lafayette, IN, USA) at a dose of 1 × 10^5^ 50% tissue culture infected infectious dose (TCID50) diluted in sterile Dulbecco’s modified Eagle medium (5 mL total volume), and the control gilts received 5 mL total volume of sterile Dulbecco’s modified Eagle medium [26,31]. Since MIA gilts exhibited decreased feed intake within 48 h [26], control gilts were fed the average consumption of the MIA gilts on the preceding day. Farrowing was induced on gestation day 113 using an intramuscular injection of 10 mg of lutalyse (Pfizer, New York, NY, USA). At birth, the pigs were given intramuscular injections of iron dextran (100 mg/pig, Butler Schein Animal Health, Dublin, OH, USA) and penicillin (60 kU/pig, Butler Schein Animal Health, Dublin, OH, USA) [26,27,29]. The pigs remained with the gilts until weaning at 22 days of age. After weaning, pigs were group-housed (four to five pigs per pen), receiving a nutritionally complete diet for growing pigs and ad libitum access to water [26,31].

Pigs were divided evenly into three postnatal stress challenge groups (STR) representing metabolic stress, inflammatory stress, and a saline-treated control at 59 days of age (five pigs per combination of MIA, STR, and sex). The experimental design is provided in Supplemental Figure S1. The metabolic stress group fasted for 24 h. On the second day (at 60 days of age) at approximately 7:00 a.m., the inflammatory stress group was intraperitoneally injected with Poly(I:C) at a dose of 1.0 mg/kg of body weight (Sigma, St. Louis, MO, USA) following published protocols [31], and the saline-treated group was injected with sterile Dulbecco’s modified Eagle medium comparable to the inflammatory stress group.

All 60 pigs were anesthetized at 60 days of age using a telazol:ketamine:xylazine drug cocktail (50 mg of tiletamine plus 50 mg of zolazepam) reconstituted with 2.5 mL ketamine (100 g/L) and 2.5 mL xylazine (100 g/L; Fort Dodge Animal Health, Fort Dodge, IA, USA) that was injected intramuscularly at a dose of 0.03 mL/kg body weight. Following anesthetization, pigs were euthanized using an intracardial injection of sodium pentobarbital (86 mg/kg body weight, Fata Plus, Vortech Pharmaceuticals, Dearborn, MI, USA). Dissected hippocampus regions were flash-frozen in dry ice and stored at −80 °C.

### 2.2. Metabolomic Sample Preparation and Analysis

Sample preparation, metabolite identifications, and profiles were obtained at the Metabolomics Center, Roy J. Carver Biotechnology Center, University of Illinois at Urbana-Champaign, using previous protocols [25,33]. Gas chromatography mass spectrometry system (Agilent Inc., Santa Clara, CA, USA) that consisted of an Agilent 7890 gas chromatograph, an Agilent 5975 MSD, and an HP 7683B autosampler. Gas chromatography was performed on a ZB-5MS (60 m × 0.32 mm I.D. and 0.25 μm film thickness) capillary column (Phenomenex, Torrance, CA, USA). The mass spectrometer was operated in positive electron impact mode (EI) at 69.9 eV ionization energy at a 30–800 *m*/*z* scan range. Metabolites were identified using a custom-built MS database that encompassed the National Institute of Standards and Technology (NIST) database with the Automatic Mass Spectral Deconvolution and Identification System (AMDIS) v2.71 (National Institute of Standards and Technology, Gaithersburg, MD, USA). All known artificial peaks were removed, and all spectra were normalized to the internal standard (hentriacontanoic acid at 10 mg/mL).

### 2.3. Statistical Analysis

The log-transformed relative abundance of the detected metabolite was described using a model that included the main effects of MIA, STR, sex, and all pairwise interactions (SAS Institute, Cary, NC, USA). *p*-values were adjusted for multiple testing using the false discovery rate (FDR) criterion [34]. Pathway enrichment analysis used annotations from the Kyoto Encyclopedia of Genes and Genomes Database (KEGG) [35] and the human metabolic pathway with the MetaboAnalyst v5 software with default specifications [36]. A canonical discriminant analysis of metabolites significantly (FDR-adjusted *p*-value < 0.05) with different abundances for MIA, STR, and the interaction between MIA and STR was conducted. Metabolites with missing values were imputed using the average log-transformed abundance of the pigs with the same MIA, STR, and sex grouping.

## 3. Results

### 3.1. Analysis of Prenatal and Postnatal Challenges

Using gas chromatography mass spectrometry, metabolites from the hippocampus of pigs were examined for the effects of MIA and STR. Pigs were exposed in utero to maternal inflammatory signals elicited from viral (PRRSV) or saline injections. Female and male pigs were subjected to one of three conditions: (a) stress of fasting for 24 h; (b) immune stress elicited by Poly(I:C) injection; or (c) saline injection. The effects of MIA, STR, sex, and interactions were examined on the abundance of 140 metabolites. The effect of sex acting alone or interacting with MIA or STR did not meet the significant threshold (FDR-adjusted *p*-value > 0.1). Similarly, eight metabolites exhibited a significant (FDR-adjusted *p*-value < 0.1) interaction between MIA and STR. Altogether, 40 metabolites had a MIA effect (FDR-adjusted *p*-value > 0.1), and 43 metabolites had a STR effect (FDR-adjusted *p*-value > 0.1). A notable finding was that 24 metabolites had MIA and STR effects (FDR-adjusted *p*-value < 0.1).

### 3.2. Pathway Enrichment

#### 3.2.1. Overrepresented Pathways

Functional analysis of the metabolites that presented MIA or STR effects detected eight enriched (FDR-adjusted *p*-value < 0.1) KEGG pathways (Table 1). Arginine biosynthesis and glutathione metabolism pathways were significantly overrepresented among the metabolites impacted by MIA or STR. The significant enrichment of the aminoacyl-tRNA biosynthesis pathway was due to the presence of multiple individual amino acids.

#### 3.2.2. Metabolites in Overrepresented Pathways

**Figure 1:**
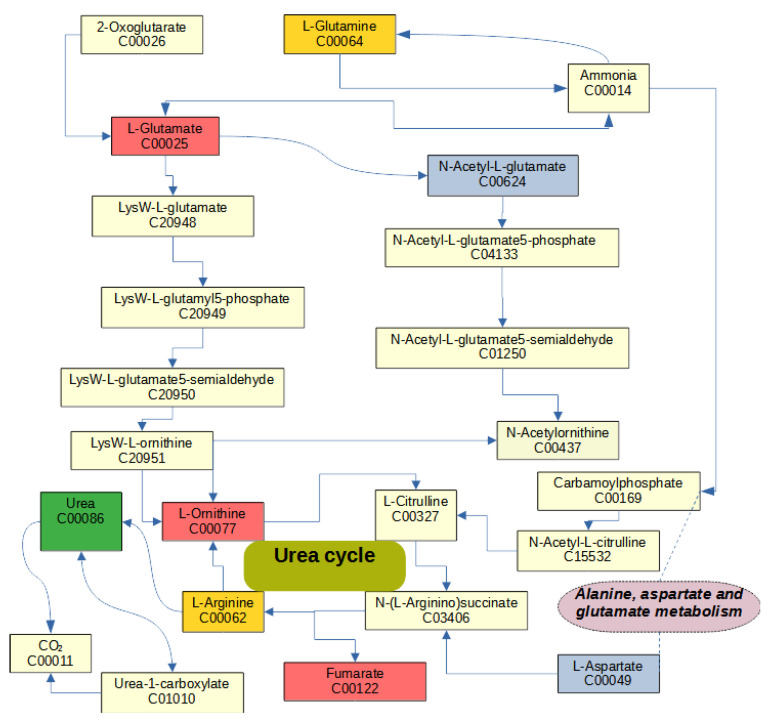
Arginine biosynthesis pathway, including metabolites that had effects (FDR-adjusted *p*-value < 0.1) on maternal immune activation (blue nodes), stress (green nodes), interaction (red nodes), and detected metabolites (gold nodes).

**Figure 2:**
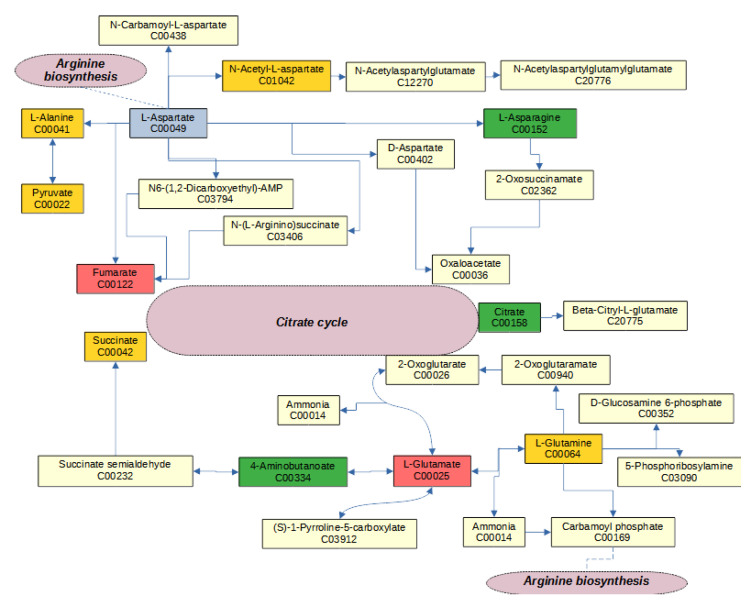
Alanine, aspartate and glutamate metabolism pathway include metabolites that had effects (FDR-adjusted *p*-value < 0.1) on maternal immune activation (blue nodes), stress (green nodes), interaction (red nodes), and detected metabolites (gold nodes).

Each enriched pathway has a different overall set of differentially abundant metabolites, and some metabolites were annotated for multiple enriched pathways. Fumarate, which presented MIA and stress effects, is annotated with three interconnected pathways: arginine biosynthesis, alanine, aspartate and glutamate metabolism, and the citric cycle (Figure 1). Arginine biosynthesis and alanine, aspartate and glutamate metabolism pathways were enriched among other metabolites that exhibited MIA and stress effects. Within the arginine biosynthesis pathway, urea cycle metabolites had MIA and stress effects (Figure 1). The alanine, aspartate and glutamate metabolism pathway (Figure 2) and the butanoate metabolism pathway were overrepresented among metabolites presenting STR effects. These metabolic pathways are connected by glutamate and 4-aminobutanoate. 

Metabolites that showed MIA effects support the enrichment of the pentose phosphate pathway. Among the previous metabolites, glyceric acid exhibited an interaction between MIA and STR. The abundance of glyceric acid was higher in pigs from PRRSV-exposed gilts relative to pigs from control gilts under Poly(I:C) or fasting stress, while this trend was not observed in saline-treated pigs. Erythrose 4-phosphate, ribose, and ribose-5-phosphate had significantly lower levels in pigs from PRRSV-exposed gilts relative to pigs from control gilts. Both erythrose 4-phosphate and ribose-5-phosphate had STR effects, and the abundance of these metabolites was lower in pigs exposed to immune stress (Poly(I:C)) relative to saline-treated or fasting pigs.

### 3.3. Metabolites Presenting Maternal Immune Activation and Stress Effects

#### 3.3.1. Interaction between MIA and Stress at 60 Days of Age

Metabolites that presented an interaction between MIA and STR (FDR-adjusted *p*-value < 0.1) are listed in Table 2. All metabolites (except for glyceric acid) were detected at lower levels in fasting pigs from PRRSV-exposed gilts MIA relative to fasting pigs from control gilts. The reverse pattern was observed for glyceric acid, which was characterized by higher levels in fasting pigs from PRRSV-exposed gilts relative to pigs from control gilts. All metabolites, except for asparagine in pigs from control gilts and leucine in pigs from PRRSV-exposed gilts, exhibited a significant STR effect within the MIA group.

#### 3.3.2. Metabolites Affected by Maternal Immune Activation or Stress

Metabolites that had one or two significant (FDR-adjusted *p*-value < 0.1) main effects included 23 metabolites presenting both MIA and STR, 12 metabolites presenting the MIA effect, and 17 metabolites presenting the STR effect. Table 3 lists the 15 metabolites that presented at least one effect (FDR-adjusted *p*-value < 0.1) and were annotated to an enriched (FDR-adjusted *p*-value < 0.1) pathway.

All 24 metabolites with a significant (FDR-adjusted *p*-value < 0.05) MIA effect had lower abundance in pigs from PRRSV-exposed gilts relative to pigs from control gilts. These metabolites are found in the arginine biosynthesis (four metabolites), glutathione metabolism (five metabolites), and purine metabolism (four metabolites) KEGG pathways. 

Overall, 24 metabolites exhibited a significant (FDR-adjusted *p*-value < 0.05) STR effect. These metabolites were found in the arginine biosynthesis (four metabolites) and glutathione metabolism (five metabolites) KEGG pathways. Urea had a significantly lower abundance in Poly(I:C)-treated pigs than both fasted and saline-injected pigs and a significantly higher abundance in fasted pigs than saline-injected pigs. Most metabolites exhibited a difference in significance between one STR group and both of the other STR groups (twelve Poly(I:C)-treated, four saline-injected, and three fasted), with no difference between the other two STR groups. The remaining four metabolites had significantly lower abundance in Poly(I:C)-treated pigs than saline-injected pigs, but neither STR group was significantly different in abundance compared to fasted pigs.

#### 3.3.3. Canonical Discriminant Analysis

**Figure 3:**
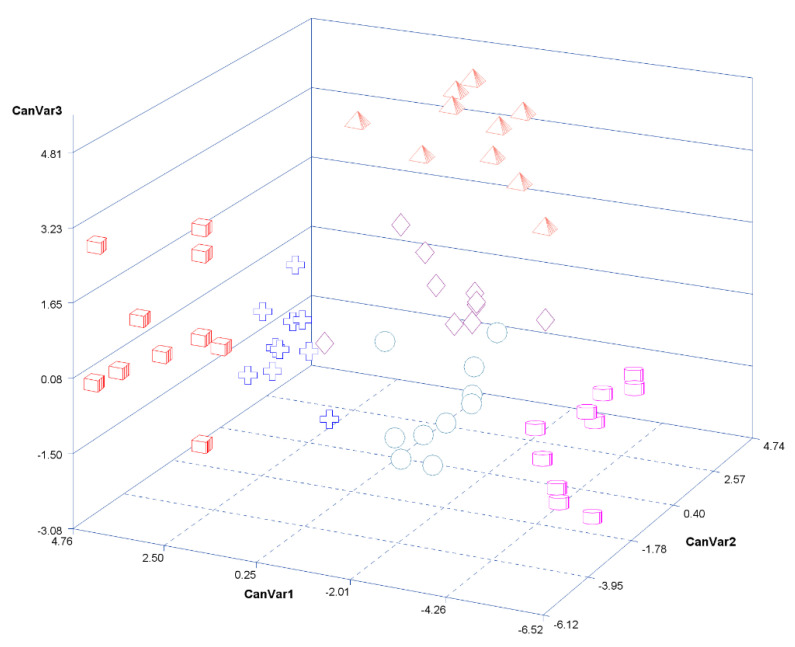
Plot of the first three canonical variables (CanVar1, CanVar2, and CanVar3) from metabolites that are significant (FDR-adjusted *p*-value < 0.05) for the effects of MIA, STR, or the interaction between MIA and STR. Red cubes denote fasted pigs from PRRSV-exposed gilts; blue crosses denote fasted pigs from control gilts; magenta cylinders denote Poly(I:C)-treated pigs from PRRSV-exposed gilts; teal circles denote Poly(I:C)-treated pigs from control gilts; light red pyramids denote saline-treated pigs from PRRSV-exposed gilts; purple diamonds denote saline-treated pigs from control gilts.

The 48 metabolites that presented significant (FDR-adjusted *p*-value < 0.05) effects of MIA, STR, or the interaction between MIA and STR were combined into three significant (*p*-value < 0.05) canonical variables. The metabolites metaglutamic acid, glycerol-3-P, glycine, inosine, lactamide, lactic acid, nicotinamide, O-phosphoethanolamine, and serine had the most extreme coefficients in the three significant canonical variables. The three significant canonical variables were able to differentiate the six combinations of MIA and STR (Figure 3). Pigs from PPRSV-exposed gilts were grouped according to the more extreme values of at least one of the three canonical variables. Fasted pigs had relatively high values for the first canonical variables for both PPRSV-exposed (red cubes) and control (blue crosses) gilts. Similarly, the Poly(I:C)-treated pigs had relatively low values for the first canonical variables for both PPRS (magenta cylinders) and control (teal circles) gilts. The saline pigs had intermediate values for the first canonical variable, with pigs from PPRS gilts (red pyramids) having relatively higher values for the third canonical variable than pigs from control (blue crosses) gilts. 

## 4. Discussion

Maternal immune activation during gestation is associated with changes in the offspring’s neurological and molecular mechanisms underlying behavioral disorders, including autism spectrum and schizophrenia, later in life [27,28,29,31,37,38]. Amino acid metabolite profiles in this study were consistent with changes in amino acids and disruption of amino acid pathways associated with stress and immune function. Disrupted amino acid metabolic pathways included alanine, aspartate and glutamate metabolism; arginine and proline metabolism; arginine biosynthesis; glycine, serine, and threonine metabolism; valine, leucine, and isoleucine biosynthesis; and glutathione metabolism. Our findings are consistent with the role of amino acids as important immunology substrates [39,40,41,42,43]. Overall, ten amino acids that exhibited significant (FDR-adjusted *p*-value < 0.1) treatment differences and two amino acid metabolism pathways were enriched among the metabolites presenting MIA or stress effects. Enrichment of the arginine biosynthesis pathway was also detected in the hippocampus of one-month old offspring of rats that were exposed to chronic restraint stress during gestation [44]. Concentrations of free amino acids were decreased in the rat brains of fetal individuals whose dams were exposed to MIA induced by Poly(I:C) compared to fetal brains from saline-treated dams [45]. Impairment of brain maturation has been associated with neurodevelopmental disorders such as autism spectrum disorders as well as future neurodegenerative processes [46,47].

The arginine pathway was overrepresented among metabolites presenting both MIA and stress effects. This finding agrees with the role of arginine as a precursor of substances that have a major role in inflammation, including nitric oxide and the polyamines putrescine, spermidine, and spermine [48]. The pathway of nitric oxide synthesis involving arginine was unlikely because citrulline was not detected in the present study. Significant effects of MIA and STR on the levels of ornithine, fumarate, and urea were observed. This is consistent with the process of arginine conversion into ornithine and urea and the subsequent conversion of ornithine into polyamines [49]. The polyamines putrescine, spermidine, and spermine were detected in this study, and the level of putrescine had a significant (FDR-adjusted *p*-value < 0.05) stress effect. Studies of the rat hippocampus support the detected effect of MIA on arginine pathway metabolites [50,51]. While Poly(I:C)-induced MIA elicited increases in the levels of putrescine and spermine in 2-day-old rats [50] and in arginine and ornithine in 3-month-old rats [51]. Likewise, the detected changes in fumarate are aligned with the proinflammatory role of fumarate and its participation in the induction of trained immunity and inhibition of histone demethylases [52,53,54].

Among the metabolites in the alanine, aspartate and glutamate metabolism pathway affected by MIA and stress, the levels of both glutamate and 4-aminobutanoate (GABA) were significantly lower in Poly(I:C)-challenged pigs relative to fasted or saline-treated 60-day-old pigs. Glutamate and GABA participate in immune responses, including as energy substrates and in cytokine production [39,40,55,56]. In addition, GABA is involved in stress responses in the hippocampus [57,58]. Consistent with our findings, enrichment of the alanine, aspartate and glutamate metabolic pathway was also reported in the hippocampus of one-month-old rats exposed to maternal stress signals during gestation in response to chronic restraint conditions [44]. GABA concentrations were decreased in rat fetal brains from Poly(I:C)-treated dams compared to fetal brains from saline-treated dams [45].

Glutamate has significant MIA and stress effects, and the products of glutamate processing, 2-oxoglutarate or 2-ketoglutaric acid, have a direct role in immune response [59]. The abundance of acetylasspartylglutamate (NAAG) and aspartate in the hippocampus of one-month-old rats born from mothers exposed to chronic restraint stress was higher than that of the control group [44]. The alanine, aspartate and glutamate metabolism pathway were enriched in the hippocampi of seven-week-old rats exposed to the stress of sleep fragmentation relative to controls [60]. Increased aspartate and glutamate levels were detected in the hippocampi of seven-week-old rats experiencing sleep fragmentation stress relative to controls [60]. Moreover, a metabolome study of the whole brain in mice concluded that aspartate deficiency influences alanine, valine, glutamate, threonine, and glycine amino acid pathways [61]. The aspartate profile was correlated with metabolites that participate in brain development and function, including choline and creatine, and in brain energy metabolism, including glucose and lactate. 

The citrate cycle pathway was not enriched among the differentially abundant metabolites, unlike a previous metabolic study of pig liver with MIA [25]. This was expected since immune cells switch from the citrate cycle and glycolysis to other energy pathways [1,59]. Individual citrate cycle pathway metabolites have important immunometabolic roles, participate in inflammatory signaling, and modulate macrophage and dendritic cell function [59,62]. Citrate, fumarate, pyruvate, and succinate were less abundant in pigs from PRRSV-exposed gilts relative to pigs from Control gilts, which is consistent with the known direct participation of citrate cycle pathway metabolites in the immune response [59,62]. 

The purine metabolism pathway was enriched among the metabolites that had MIA and stress effects. Purines provide stress protection, participate in immune cell development, and modulate inflammation [6,63]. Purine-based metabolites adenine, guanine, inosine, and xanthine presented significant MIA and stress effects, while hypoxanthine had significant MIA effects. In addition, the abundance of other metabolites in the purine pathway changed across MIA groups (i.e., oxalic acid) and stress (i.e., urea). Xanthine oxidoreductase is involved in many biological processes that participate in the formation of important metabolites [64], including the conversion of hypoxanthine to xanthine and the conversion of xanthine to uric acid, which is eventually converted to urea. Aligned with our findings, the abundance of urea was lower and the abundance of hypoxanthine was higher in the hippocampus of one-month-old rats born from mothers exposed to chronic restraint stress compared to the control group [44]. Similarly, increased hypoxanthine was observed in the hippocampi of seven-week-old rats that experienced stress due to sleep fragmentation relative to controls [60].

The glutathione metabolism pathway was enriched among metabolites presenting significant MIA and stress effects. In addition to differentially abundant metabolites shared with the arginine pathway (i.e., glutamate, ornithine, and putrescine), MIA and stress had a significant effect on glutathione and 5-oxoproline. The abundance of the previous metabolites was lower in stressed pigs or pigs from PRRSV-exposed gilts relative to saline-treated pigs from Control gilts. The effects detected in the present study are aligned with the known roles of glutathione in cytokine production and immune response [65], and lower levels of glutathione have a negative impact on the immune response [66].

Enrichment of the pentose phosphate pathway affected by MIA is consistent with the association between this pathway and pathogen infection. The pentose phosphate pathway produces the essential electron donor nicotinamide adenine dinucleotide phosphate (NADPH), which participates in the generation of reactive oxygen species and antioxidants [1]. Neutrophils switch to the pentose phosphate pathway for a rapid increase in the production of superoxide ion that supports the response against pathogenic infection [3]. The abundance of erythrose 4-phosphate and ribose-5-phosphate, two metabolites specifically found in the pentose phosphate pathway, was significantly lower in PRRSV-exposed pigs relative to pigs from Control gilts. These profiles suggest that MIA may hinder the metabolic readiness of the hippocampus to mount a response to inflammatory signals later in life. The metabolic reprogramming elicited by MIA is consistent with the effects of MIA on the transcriptome and cell type that persist after infection [67].

The interaction pattern was characterized by differential abundance (*p*-value < 0.01) between stressors within pigs from PRRSV-exposed gilts and between prenatal activation groups within fasted pigs. Among pigs from PRRSV-exposed gilts, fasted pigs had the lowest metabolite levels, followed by Poly(I:C)-challenged pigs, and saline-treated pigs had the highest levels. Within the pigs from control gilts, there was no difference between Poly(I:C)-challenged and saline-treated pigs, and two metabolites had significantly different abundances between fasted and saline-treated pigs. In contrast, within pigs from PRRSV-exposed gilts, five metabolites had significantly different abundances between fasting and saline-treated pigs, and one metabolite had significantly different abundances between Poly(I:C)-challenged and saline-treated pigs. 

A notable finding is that many of the enriched pathways associated with prenatal inflammatory conditions (i.e., MIA) in the hippocampus have been associated with addiction, psychostimulant-elicited neuroinflammation, and neurogenesis impairment [68]. Aligned with the significant over-representation of the arginine biosynthesis metabolism pathway in the hippocampus of pigs exposed to MIA, rats administered cocaine presented increased levels of arginine in the hippocampus [69]. MIA and heroin exposure resulted in enrichment of the glutathione metabolism pathway and changes in glutamate levels in the brains of mice subcutaneously injected with heroin [70]. The changes in glutamic acid levels detected in the present study are consistent with the decreased levels in the cerebellum of rats exposed to synthetic cannabinoids [71]. The changes in the levels of purine nucleotides and secondary metabolites (i.e., hypoxanthine) in association with prenatal and postnatal challenges detected in the present study have also been reported in studies of the effects of heroin exposure in the brains of mice and rats [70,72,73].

These results support the pertinence of the double-hit hypothesis on metabolic pathways in the hippocampus. Metabolic profile changes suggest that, in addition to their independent effects, MIA and metabolic or inflammatory stressors later in life have interacting modes of action on hippocampal metabolites. The interaction between MIA and stress for some metabolites, including asparagine, glutamic acid, glycine, and serine, also supported the double-hit hypothesis. Other metabolites that had significant MIA and stressor effects while the interaction did not reach a similar level presented a similar pattern, with pigs from PRRSV-exposed gilts presenting a higher response to the stressors at 60 days of age. The influence of the double-hit hypothesis is evident in the canonical discrimination analysis, where pigs from PPRS-exposed gilts were more extreme than the pigs from control gilts for each stress grouping. Differences between groups with metabolites with a significant interaction between MIA and stress indicate that the type of stress can enhance the double-hit effect.

The detected changes in metabolites associated with stressors further the understanding of disruptions in input and output signals that are the foundation of the hippocampal function as a hub for integrating metabolic signals with cognitive processes [10]. Many of the metabolites (e.g., glutamate, ornithine, and putrescine) impacted by MIA and stress participate in multiple biochemical pathways, including the arginine, glutathione, and alanine, aspartate and glutamate pathways. This finding has multiple implications, including that the effects of MIA and stressors impact various pathways with different degrees of overlap. The disruption of the previous pathways may underlie reports of behavioral and physiological changes in 60-day-old pigs exposed to MIA and challenged with Poly(I:C). The studied pre- and post-natal challenges elicited significant changes in peripheral stress, biochemical and immune biomarkers [24,37], and pig behavior [31]. Another implication is that therapies addressing one metabolite disruption will impact multiple possible pathways, including the inhibitory and feed-forward effects of the hippocampus and HPA axis function, including the onset and termination of responses to stress and behaviors [74].

## 5. Conclusions

Hippocampi metabolites measured at 60 days of age were significantly impacted by prenatal and postnatal stressors in a pig model of MIA. For some metabolites (e.g., fumarate, hypoxanthine, and ornithine), the prenatal and postnatal challenges studied acted in an independent manner, and several metabolites exhibited persistent effects from MIA. A significant interaction between MIA and stressors later in life for other metabolites (e.g., asparagine, glutamic acid, glycine, and serine) supported the double-hit hypothesis that MIA amplifies the impact of stressors later in life. The profile of some metabolites supported an additive effect where the influence of stressors later in life was enhanced by MIA. Many metabolites perturbed by prenatal and postnatal stressors are directly associated with both immune and stress responses. These metabolites also appear in multiple biochemical pathways associated with immune and stress responses. The effects of MIA and stressors were more prevalent in the arginine, pentose phosphate, and alanine, aspartate and glutamate metabolic pathways. The overrepresentation of the arginine pathway among the metabolite profiles impacted by MIA and stress could be associated with the biosynthesis of polyamines, in particular the significant change in putrescine, which participates in the immune response. Disruption of the amino acid metabolic pathway and metabolites by prenatal inflammatory signals was also observed in drug abuse studies. Our results demonstrate that metabolomic analysis of the hippocampus and other brain structures can lay the foundation for understanding the mechanisms underlying the long-lasting effect of MIA and its interplay with stressors later in life on health and behavior.

## Figures and Tables

**Table 1:** Number of metabolites (Hits) and significant (FDR-adjusted *p*-value < 0.1) enriched KEGG pathways among the metabolites presenting maternal immune activation (MIA) or stress (STR) effects.

KEGG Pathway	MIA		STR	
	Hits	Impact ^a^	Hits	Impact
Amino acid metabolism				
Alanine, aspartate and glutamate metabolism	3	0.423	5	0.286 **
Arginine biosynthesis	5	0.178 ***	4	0.178 **
Carbohydrate metabolism				
Butanoate metabolism	1	0.000	3	0.032 *
Glyoxylate and dicarboxylate metabolism	4	0.228 *	2	0.032
Pentose phosphate pathway	4	0.176 **	2	0.176
Metabolism of other amino acids				
Glutathione metabolism	5	0.371 **	5	0.290 **
Nucleotide metabolism				
Purine metabolism	7	0.128 **	7	0.111 **
Translation				
Aminoacyl-tRNA biosynthesis	7	0.167 ***	6	0.000 **

^a^ Pathway Impact Score; *** pathways significantly (FDR-adjusted *p*-value < 0.01) enriched; ** pathways significantly (FDR-adjusted *p*-value < 0.05) enriched; * pathways significantly (FDR-adjusted *p*-value < 0.10) enriched.

**Table 2:** Log-transformed normalized abundance (± standard error) of the metabolites exhibiting a maternal immune activation-by-stress interaction effect (FDR-adjusted *p*-value < 0.1).

	NP ^1^	Control ^2^			PRRSV		
Metabolite		Saline ^3^	Fasted	Poly(I:C)	Saline	Fasted	Poly(I:C)
Asparagine	2	3.5 ± 0.2 ^a^	3.5 ± 0.2 ^a^	3.2 ± 0.2 ^ab^	3.8 ± 0.2 ^a^	2.6 ± 0.2 ^b^	3.4 ± 0.2 ^a^
Glutamic acid	8	10.1 ± 0.1 ^ab^	10.3 ± 0.1 ^a^	10.0 ± 0.1 ^abc^	10.2 ± 0.1 ^a^	9.8 ± 0.1 ^c^	9.8 ± 0.1 ^bc^
Glyceric acid	3	2.2 ± 0.1 ^ab^	1.9 ± 0.1 ^ab^	1.6 ± 0.1 ^b^	2.0 ± 0.1 ^ab^	2.5 ± 0.1 ^a^	2.2 ± 0.1 ^ab^
Glycine	5	8.6 ± 0.1 ^ab^	8.6 ± 0.1 ^a^	8.4 ± 0.1 ^abc^	8.5 ± 0.1 ^ab^	8.1 ± 0.1 ^c^	8.3 ± 0.1 ^bc^
Inositol #2	0	4.9 ± 0.2 ^ab^	5.0 ± 0.2 ^a^	4.3 ± 0.2 ^b^	5.1 ± 0.2 ^a^	4.3 ± 0.2 ^b^	4.6 ± 0.2 ^ab^
Inositol #3	0	1.3 ± 0.2 ^b^	1.9 ± 0.2 ^a^	1.3 ± 0.2 ^ab^	1.8 ± 0.2 ^ab^	1.1 ± 0.2 ^b^	1.3 ± 0.2 ^ab^
Leucine	2	5.1 ± 0.1 ^b^	5.7 ± 0.1 ^a^	5.1 ± 0.1 ^b^	5.0 ± 0.1 ^b^	4.8 ± 0.1 ^b^	4.8 ± 0.1 ^b^
Serine	3	8.2 ± 0.1 ^ab^	8.5 ± 0.1 ^a^	8.1 ± 0.1 ^abc^	8.3 ± 0.1 ^ab^	7.8 ± 0.1 ^c^	8.0 ± 0.1 ^bc^

^1^ NP: Number of pathways in the Kyoto Encyclopedia of Genes and Genomes Database that include that metabolite. ^2^ Maternal immune activation groups: Control = pigs from control gilts; PRRSV = pigs from PRRSV-exposed gilts. ^3^ Stress groups: saline; fasted for 24 h; exposed to Poly(I:C) immune challenge. ^abc^ Letters within rows denote differences between consecutive levels (Tukey-adjusted *p*-value < 0.05) within MIA or Stress groups.

**Table 3:** Log-transformed normalized abundance (±standard error) of the metabolites exhibiting an effect (FDR-adjusted *p*-value < 0.1) of maternal immune activation or stress at 60 days of age and present in at least one overrepresented pathway.

Metabolite ^1^	MIA ^2^		STR ^3^		
	Control	PRRSV	Saline	Fasted	Poly(I:C)
3-HBA	3.08 ± 0.08	2.94 ± 0.08	2.86 ± 0.10 ^b^	3.39 ± 0.10 ^a^	2.79 ± 0.10 ^b^
Adenine	5.36 ± 0.11 ^a^	4.97 ± 0.11 ^b^	5.45 ± 0.13 ^a^	5.21 ± 0.13 ^ab^	4.83 ± 0.13 ^b^
AMP	6.81 ± 0.09 ^a^	6.37 ± 0.09 ^b^	6.82 ± 0.11 ^a^	6.66 ± 0.11 ^ab^	6.28 ± 0.11 ^b^
Aspartic acid	8.81 ± 0.05 ^a^	8.56 ± 0.05 ^b^	8.75 ± 0.07	8.72 ± 0.07	8.57 ± 0.07
Fumaric acid	5.38 ± 0.06 ^a^	5.07 ± 0.06 ^b^	5.40 ± 0.07 ^a^	5.25 ± 0.07 ^ab^	5.02 ± 0.07 ^b^
Guanine	3.09 ± 0.04 ^a^	2.94 ± 0.05 ^b^	3.16 ± 0.05 ^a^	2.94 ± 0.06 ^b^	2.94 ± 0.06 ^b^
Hypoxanthine	6.08 ± 0.09 ^a^	5.71 ± 0.09 ^b^	5.97 ± 0.11	5.92 ± 0.11	5.79 ± 0.11
Inosine	8.36 ± 0.07 ^a^	7.99 ± 0.07 ^b^	8.35 ± 0.09 ^a^	8.19 ± 0.09 ^ab^	7.98 ± 0.09 ^b^
Lysine	5.52 ± 0.06	5.43 ± 0.06	5.50 ± 0.07 ^a^	5.70 ± 0.07 ^a^	5.23 ± 0.07 ^b^
Ornithine	4.00 ± 0.07 ^a^	3.70 ± 0.07 ^b^	4.06 ± 0.08 ^a^	3.70 ± 0.08 ^b^	3.79 ± 0.08 ^ab^
Putrescine	3.47 ± 0.10	3.41 ± 0.10	3.67 ± 0.12 ^a^	3.50 ± 0.12 ^ab^	3.14 ± 0.12 ^b^
pGlutamic acid	10.23 ± 0.05 ^a^	10.01 ± 0.05 ^b^	10.25 ± 0.06 ^a^	10.12 ± 0.06 ^a^	9.99 ± 0.06 ^b^
Ribose	4.74 ± 0.11 ^a^	4.27 ± 0.11 ^b^	4.57 ± 0.13	4.61 ± 0.13	4.33 ± 0.13
Urea	8.16 ± 0.09	8.11 ± 0.09	8.08 ± 0.10 ^b^	8.61 ± 0.10 ^a^	7.71 ± 0.10 ^c^
Valine	5.48 ± 0.08	5.33 ± 0.08	5.35 ± 0.09 ^b^	5.79 ± 0.09 ^a^	5.09 ± 0.09 ^b^

^1^ Metabolite: 3-HBA = 3-Hydroxybutanoic acid; AMP= Adenosine-5-Monophosphate; pGlutamic acid =Pyroglutamic acid. ^2^ Maternal immune activation (MIA) groups: Control = pigs from control gilts; PRRSV = pigs from PRRSV-exposed gilts. ^3^ Stress (STR) groups: saline; fasted for 24 h; exposed to Poly(I:C) immune challenge. ^abc^ Letters within rows denote differences between consecutive levels (Tukey-adjusted *p*-value < 0.05) within MIA or STR groups.

## Data Availability

Data is available from the corresponding author on request. Data is not publicly available due to privacy.

## Supplementary Materials

The following supporting information can be downloaded at: https://www.mdpi.com/article/10.3390/metabo13080881/s1, Figure S1: Experimental design scheme.

## References

[1] O’Neill L.A., Kishton R.J., Rathmell J. (2016). A guide to immunometabolism for immunologists. Nat. Rev. Immunol..

[2] Loftus R.M., Finlay D.K. (2016). Immunometabolism: Cellular Metabolism Turns Immune Regulator. J. Biol. Chem..

[3] Britt E.C., Lika J., Giese M.A., Schoen T.J., Seim G.L., Huang Z., Lee P.Y., Huttenlocher A., Fan J. (2022). Switching to the cyclic pentose phosphate pathway powers the oxidative burst in activated neutrophils. Nat. Metab..

[4] O’Neill L.A., Pearce E.J. (2015). Immunometabolism governs dendritic cell and macrophage function. J. Exp. Med..

[5] Antonioli L., Fornai M., Blandizzi C., Pacher P., Haskó G. (2019). Adenosine signaling and the immune system: When a lot could be too much. Immunol. Lett..

[6] Pasquini S., Contri C., Borea P.A., Vincenzi F., Varani K. (2021). Adenosine and Inflammation: Here, There and Everywhere. Int. J. Mol. Sci..

[7] Usuda K., Kawase T., Shigeno Y., Fukuzawa S., Fujii K., Zhang H., Tsukahara T., Tomonaga S., Watanabe G., Jin W. (2018). Hippocampal metabolism of amino acids by L-amino acid oxidase is involved in fear learning and memory. Sci. Rep..

[8] Leuner B., Gould E. (2010). Structural Plasticity and Hippocampal Function. Annu. Rev. Psychol..

[9] Maynard T., Sikich L., Lieberman J.A., LaMantia A.-S. (2001). Neural Development, Cell-Cell Signaling, and the “Two-Hit” Hypothesis of Schizophrenia. Schizophr. Bull..

[10] Tingley D., McClain K., Kaya E., Carpenter J., Buzsáki G. (2021). A metabolic function of the hippocampal sharp wave-ripple. Nature.

[11] Lumertz F.S., Kestering-Ferreira E., Orso R., Creutzberg K.C., Tractenberg S.G., Stocchero B.A., Viola T.W., Grassi-Oliveira R. (2022). Effects of early life stress on brain cytokines: A systematic review and meta-analysis of rodent studies. Neurosci. Biobehav. Rev..

[12] Sheng J.A., Bales N.J., Myers S.A., Bautista A.I., Roueinfar M., Hale T.M., Handa R.J. (2020). The Hypothalamic-Pituitary-Adrenal Axis: Development, Programming Actions of Hormones, and Maternal-Fetal Interactions. Front. Behav. Neurosci..

[13] Bellavance M.-A., Rivest S. (2014). The HPA â€“ Immune Axis and the Immunomodulatory Actions of Glucocorticoids in the Brain. Front. Immunol..

[14] Leone P., Mincheva G., Balzano T., Malaguarnera M., Felipo V., Llansola M. (2022). Rifaximin Improves Spatial Learning and Memory Impairment in Rats with Liver Damage-Associated Neuroinflammation. Biomedicines.

[15] Odorizzi P.M., Feeney M.E. (2016). Impact of In Utero Exposure to Malaria on Fetal T Cell Immunity. Trends Mol. Med..

[16] Prins J.R., Eskandar S., Eggen B.J., Scherjon S.A. (2018). Microglia, the missing link in maternal immune activation and fetal neurodevelopment; and a possible link in preeclampsia and disturbed neurodevelopment?. J. Reprod. Immunol..

[17] Poletto R., Steibel J., Siegford J., Zanella A. (2006). Effects of early weaning and social isolation on the expression of glucocorticoid and mineralocorticoid receptor and 11β-hydroxysteroid dehydrogenase 1 and 2 mRNAs in the frontal cortex and hippocampus of piglets. Brain Res..

[18] Han V.X., Patel S., Jones H.F., Dale R.C. (2021). Maternal immune activation and neuroinflammation in human neurodevelopmental disorders. Nat. Rev. Neurol..

[19] Boulanger-Bertolus J., Pancaro C., Mashour G.A. (2018). Increasing Role of Maternal Immune Activation in Neurodevelopmental Disorders. Front. Behav. Neurosci..

[20] Bayer T.A., Falkai P., Maier W. (1999). Genetic and non-genetic vulnerability factors in schizophrenia: The basis of the “Two hit hypothesis”. J. Psychiatr. Res..

[21] Walker A.K., Nakamura T., Byrne R.J., Naicker S., Tynan R.J., Hunter M., Hodgson D.M. (2009). Neonatal lipopolysaccharide and adult stress exposure predisposes rats to anxiety-like behaviour and blunted corticosterone responses: Implications for the double-hit hypothesis. Psychoneuroendocrinology.

[22] Imanaka A., Morinobu S., Toki S., Yamawaki S. (2006). Importance of early environment in the development of post-traumatic stress disorder-like behaviors. Behav. Brain Res..

[23] Giovanoli S., Meyer U. (2013). Response to Comment on “Stress in Puberty Unmasks Latent Neuropathological Consequences of Prenatal Immune Activation in Mice”. Science.

[24] Rymut H.E., Rund L.A., Bolt C.R., Villamil M.B., Southey B.R., Johnson R.W., Rodriguez-Zas S.L. (2021). The Combined Effect of Weaning Stress and Immune Activation during Pig Gestation on Serum Cytokine and Analyte Concentrations. Animals.

[25] Southey B.R., Bolt C.R., Rymut H.E., Keever M.R., Ulanov A.V., Li Z., Rund L.A., Johnson R.W., Rodriguez-Zas S.L. (2021). Impact of Weaning and Maternal Immune Activation on the Metabolism of Pigs. Front. Mol. Biosci..

[26] Keever M.R., Zhang P., Bolt C.R., Antonson A.M., Rymut H.E., Caputo M.P., Houser A.K., Hernandez A.G., Southey B.R., Rund L.A. (2020). Lasting and Sex-Dependent Impact of Maternal Immune Activation on Molecular Pathways of the Amygdala. Front. Neurosci..

[27] Keever-Keigher M.R., Zhang P., Bolt C.R., Rymut H.E., Antonson A.M., Caputo M.P., Houser A.K., Hernandez A.G., Southey B.R., Rund L.A. (2021). Interacting impact of maternal inflammatory response and stress on the amygdala transcriptome of pigs. G3.

[28] Southey B.R., Keever-Keigher M.R., Rymut H.E., Rund L.A., Johnson R.W., Rodriguez-Zas S.L. (2021). Disruption of Alternative Splicing in the Amygdala of Pigs Exposed to Maternal Immune Activation. Immuno.

[29] Rymut H.E., Rund L.A., Southey B.R., Johnson R.W., Rodriguez-Zas S.L. (2022). Terpenoid Backbone Biosynthesis among Pig Hippocampal Pathways Impacted by Stressors. Genes.

[30] Rodriguez-Zas S.L., Southey B.R., Rymut H.E., Rund L.A., Johnson R.W. (2022). Hippocampal Changes Elicited by Metabolic and Inflammatory Stressors following Prenatal Maternal Infection. Genes.

[31] Rymut H.E., Bolt C.R., Caputo M.P., Houser A.K., Antonson A.M., Zimmerman J.D., Villamil M.B., Southey B.R., Rund L.A., Johnson R.W. (2020). Long-Lasting Impact of Maternal Immune Activation and Interaction with a Second Immune Challenge on Pig Behavior. Front. Veter Sci..

[32] Geisert R.D., Lucy M.C., Skinner M.K. (2018). Pig. Encyclopedia of Reproduction.

[33] Wu H., Southam A.D., Hines A., Viant M.R. (2008). High-throughput tissue extraction protocol for NMR- and MS-based metabolomics. Anal. Biochem..

[34] Benjamini Y., Hochberg Y. (1995). Controlling the False Discovery Rate: A Practical and Powerful Approach to Multiple Testing. J. R. Stat. Soc. Ser. B Methodol..

[35] Kanehisa M., Goto S. (2000). KEGG: Kyoto Encyclopedia of Genes and Genomes. Nucleic Acids Res..

[36] Pang Z., Chong J., Zhou G., de Lima Morais D.A., Chang L., Barrette M., Gauthier C., Jacques P.-É., Li S., Xia J. (2021). MetaboAnalyst 5.0: Narrowing the gap between raw spectra and functional insights. Nucleic Acids Res..

[37] Rymut H.E., Rund L.A., Bolt C.R., Villamil M.B., Bender D.E., Southey B.R., Johnson R.W., Rodriguez-Zas S.L. (2021). Biochemistry and Immune Biomarkers Indicate Interacting Effects of Pre- and Postnatal Stressors in Pigs across Sexes. Animals.

[38] Southey B.R., Zhang P., Keever M.R., Rymut H.E., Johnson R.W., Sweedler J.V., Rodriguez-Zas S.L. (2021). Effects of maternal immune activation in porcine transcript isoforms of neuropeptide and receptor genes. J. Integr. Neurosci..

[39] Li P., Yin Y.-L., Li D., Kim S.W., Wu G. (2007). Amino acids and immune function. Br. J. Nutr..

[40] Sikalidis A.K. (2015). Amino Acids and Immune Response: A Role for Cysteine, Glutamine, Phenylalanine, Tryptophan and Arginine in T-cell Function and Cancer?. Pathol. Oncol. Res..

[41] Miyajima M. (2020). Amino acids: Key sources for immunometabolites and immunotransmitters. Int. Immunol..

[42] Kelly B., Pearce E.L. (2020). Amino Assets: How Amino Acids Support Immunity. Cell Metab..

[43] Hamill M.J., Afeyan R., Chakravarthy M.V., Tramontin T. (2020). Endogenous Metabolic Modulators: Emerging Therapeutic Potential of Amino Acids. iScience.

[44] Zhang H., He W., Huang Y., Zeng Z., Yang X., Huang H., Wen J., Cao Y., Sun H. (2019). Hippocampal metabolic alteration in rat exhibited susceptibility to prenatal stress. J. Affect. Disord..

[45] McColl E.R., Piquette-Miller M. (2019). Poly(I:C) alters placental and fetal brain amino acid transport in a rat model of maternal immune activation. Am. J. Reprod. Immunol..

[46] Nguyen Y.T.K., Ha H.T.T., Nguyen T.H., Nguyen L.N. (2021). The role of SLC transporters for brain health and disease. Cell. Mol. Life Sci..

[47] Ouellette J., Lacoste B. (2021). From Neurodevelopmental to Neurodegenerative Disorders: The Vascular Continuum. Front. Aging Neurosci..

[48] Morris S.M. (2016). Arginine Metabolism Revisited. J. Nutr..

[49] Proietti E., Rossini S., Grohmann U., Mondanelli G. (2020). Polyamines and Kynurenines at the Intersection of Immune Modulation. Trends Immunol..

[50] Jing Y., Zhang H., Wolff A.R., Bilkey D.K., Liu P. (2013). Altered arginine metabolism in the hippocampus and prefrontal cortex of maternal immune activation rat offspring. Schizophr. Res..

[51] Zhang J., Jing Y., Zhang H., Bilkey D.K., Liu P. (2018). Effects of maternal immune activation on brain arginine metabolism of postnatal day 2 rat offspring. Schizophr. Res..

[52] Fraschilla I., Amatullah H., Jeffrey K.L. (2022). One genome, many cell states: Epigenetic control of innate immunity. Curr. Opin. Immunol..

[53] Choi I., Son H., Baek J.-H. (2021). Tricarboxylic Acid (TCA) Cycle Intermediates: Regulators of Immune Responses. Life.

[54] Arts R.J., Novakovic B., ter Horst R., Carvalho A., Bekkering S., Lachmandas E., Rodrigues F., Silvestre R., Cheng S.-C., Wang S.-Y. (2016). Glutaminolysis and Fumarate Accumulation Integrate Immunometabolic and Epigenetic Programs in Trained Immunity. Cell Metab..

[55] Cruzat V., Macedo Rogero M., Keane K.N., Curi R., Newsholme P. (2018). Glutamine: Metabolism and Immune Function, Supplementation and Clinical Translation. Nutrients.

[56] Jin Z., Mendu S.K., Birnir B. (2013). GABA is an effective immunomodulatory molecule. Amino Acids.

[57] Czeh B., Varga Z.K., Henningsen K., Kovacs G.L., Miseta A., Wiborg O. (2015). Chronic stress reduces the number of GABAergic interneurons in the adult rat hippocampus, dorsal-ventral and region-specific differences. Hippocampus.

[58] Dolfen N., Veldman M.P., Gann M.A., von Leupoldt A., Puts N.A.J., Edden R.A.E., Mikkelsen M., Swinnen S., Schwabe L., Albouy G. (2021). A role for GABA in the modulation of striatal and hippocampal systems under stress. Commun. Biol..

[59] Zasłona Z., O’neill L.A. (2020). Cytokine-like Roles for Metabolites in Immunity. Mol. Cell.

[60] Yoon D.W., Kwon H.N., Jin X., Kim J.K., Lee S.K., Park S., Yun C.-H., Shin C. (2019). Untargeted metabolomics analysis of rat hippocampus subjected to sleep fragmentation. Brain Res. Bull..

[61] Grimaldi M., Marino C., Buonocore M., Santoro A., Sommella E., Merciai F., Salviati E., De Rosa A., Nuzzo T., Errico F. (2021). Prenatal and Early Postnatal Cerebral d-Aspartate Depletion Influences l-Amino Acid Pathways, Bioenergetic processes, and Developmental Brain Metabolism. J. Proteome Res..

[62] Williams N.C., O’neill L.A.J. (2018). A Role for the Krebs Cycle Intermediate Citrate in Metabolic Reprogramming in Innate Immunity and Inflammation. Front. Immunol..

[63] Koch-Nolte F., Dahl G. (2019). Purine Release, Metabolism, and Signaling in the Inflammatory Response. Annu. Rev. Immunol..

[64] Bortolotti M., Polito L., Battelli M.G., Bolognesi A. (2021). Xanthine oxidoreductase: One enzyme for multiple physiological tasks. Redox Biol..

[65] Wu G., Lupton J.R., Turner N.D., Fang Y.-Z., Yang S. (2004). Glutathione Metabolism and Its Implications for Health. J. Nutr..

[66] Rodrigues C., Percival S.S. (2019). Immunomodulatory Effects of Glutathione, Garlic Derivatives, and Hydrogen Sulfide. Nutrients.

[67] Canales C.P., Estes M.L., Cichewicz K., Angara K., Aboubechara J.P., Cameron S., Prendergast K., Su-Feher L., Zdilar I., Kreun E.J. (2021). Sequential perturbations to mouse corticogenesis following in utero maternal immune activation. Elife.

[68] Asser A., Taba P. (2015). Psychostimulants and Movement Disorders. Front. Neurol..

[69] Li Y., Wang X., Ge S.-N., Wang X.-L. (2022). Alterations in Neurotransmitters Targeted Metabolomics from the Key Nuclei of Brain Reward Circuits in Cocaine-Induced Behavioral Sensitization for Self-Administering Rats. SSRN.

[70] Li L., Cao H., Li J., Kuang H., Zhou Z., Wang Q. (2022). Metabolomics analysis reveals how water extract of Gastrodia elata helps against heroin addiction. Pharmacol. Res. Mod. Chin. Med..

[71] Zaitsu K., Hayashi Y., Suzuki K., Nakayama H., Hattori N., Takahara R., Kusano M., Tsuchihashi H., Ishii A. (2015). Metabolome disruption of the rat cerebrum induced by the acute toxic effects of the synthetic cannabinoid MAM-2201. Life Sci..

[72] Li K., He H.-T., Li H.-M., Liu J.-K., Fu H.-Y., Hong M. (2011). Heroin affects purine nucleotides metabolism in rat brain. Neurochem. Int..

[73] Yang Y.-D., Zhang J.-Z., Sun C., Yu H.-M., Li Q., Hong M. (2006). Heroin affects purine nucleotides catabolism in rats in vivo. Life Sci..

[74] Jacobson L., Sapolsky R. (1991). The Role of the Hippocampus in Feedback Regulation of the Hypothalamic-Pituitary-Adrenocortical Axis. Endocr. Rev..

