# Supplementary material for: Influence of Maternal Immune Activation and Stressors on the Hippocampal Metabolome
Source: Metabolites. 2023 Jul 25;13(8):881. doi: 10.3390/metabo13080881 (PMC10456262; doi:10.3390/metabo13080881)
Supplement: Supplementary file 1 [file metabolites-13-00881-s001.zip › metabolites-2478517-supplementary.pdf]

Supplemental Materials

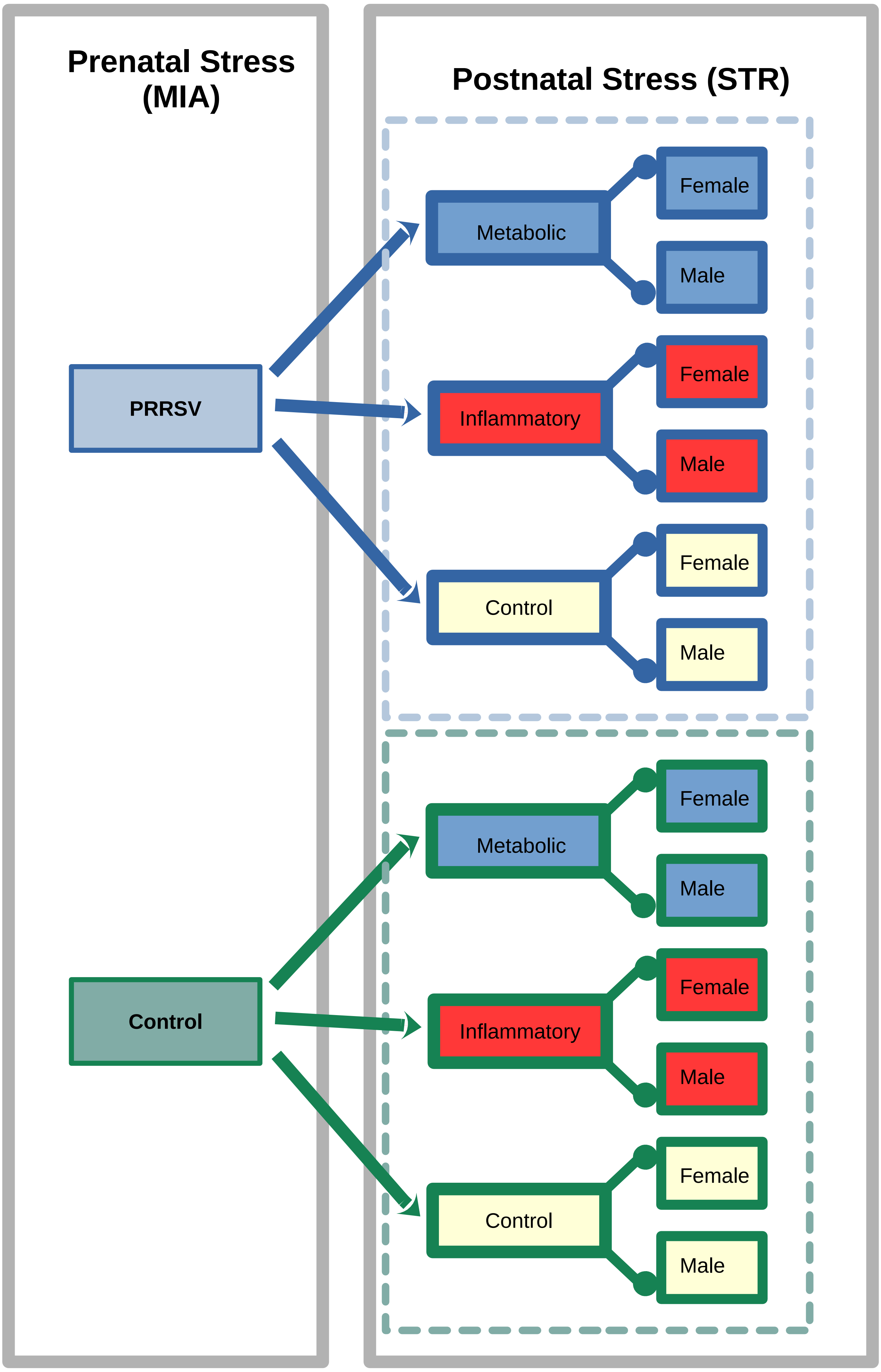

Supplemental Figure S1. Experimental design scheme. Prenatal stress, maternal immune activation (MIA) was elicited by the porcine reproductive and respiratory syndrome virus (PRRSV) or control to pregnant gilts. Postnatal stress involved metabolic stress (fasting pigs for 1 day), inflammatory stress (injected with Poly(I:C)), and saline-treated control.
